# An Updated Comprehensive Review on Diseases Associated with Nephrotic Syndromes

**DOI:** 10.3390/biomedicines12102259

**Published:** 2024-10-04

**Authors:** Ralph Wendt, Alina Sobhani, Paul Diefenhardt, Moritz Trappe, Linus Alexander Völker

**Affiliations:** 1 Department of Nephrology, Hospital St. Georg Leipzig, Delitzscher Str. 141, 04129 Leipzig, Germany; 2 Department II of Internal Medicine, Center for Molecular Medicine Cologne, Faculty of Medicine, University Hospital Cologne, University of Cologne, 50937 Cologne, Germany; alina.sobhani1@uk-koeln.de (A.S.); paul.diefenhardt@uk-koeln.de (P.D.); moritz.trappe@uk-koeln.de (M.T.); linus.voelker@uk-koeln.de (L.A.V.); 3 Cologne Cluster of Excellence on Cellular Stress Responses in Ageing-Associated Diseases, 50923 Cologne, Germany

**Keywords:** nephrotic syndrome, minimal change disease, FSGS, membranous nephropathy, MGRS, IgAN, DKD, MPGN, edema, proteinuria, PLA2R, nephrin

## Abstract

There have been exciting advances in our knowledge of primary glomerular diseases and nephrotic syndromes in recent years. Beyond the histological pattern from renal biopsy, more precise phenotyping of the diseases and the use of modern nephrogenetics helps to improve treatment decisions and sometimes also avoid unnecessary exposure to potentially toxic immunosuppression. New biomarkers have led to easier and more accurate diagnoses and more targeted therapeutic decisions. The treatment landscape is becoming wider with a pipeline of promising new therapeutic agents with more sophisticated approaches. This review focuses on all aspects of entities that are associated with nephrotic syndromes with updated information on recent advances in each field. This includes podocytopathies (focal segmental glomerulosclerosis and minimal-change disease), membranous nephropathy, membranoproliferative glomerulonephritis, IgA nephropathy, fibrillary glomerulonephritis, amyloidosis, and monoclonal gammopathy of renal significance in the context of the nephrotic syndrome, but also renal involvement in systemic diseases, diabetic nephropathy, and drugs that are associated with nephrotic syndromes.

## 1. Introduction and General Aspects of Nephrotic Syndrome

Nephrotic syndrome is characterized by certain clinical and laboratory features of kidney disease. It is defined by proteinuria greater than 3.5 g/24 h, hypoalbuminemia, peripheral edema, and hyperlipidemia. Nephrotic syndrome is not a disease itself but a consequence or a manifestation of an underlying kidney disease.

Overall, nephrotic syndromes are rare. The annual incidence of nephrotic syndrome is approximately 3 cases per 100,000 adults [1]. Because of the potential serious complications, any patient presenting with a new edema of an unknown cause should always be evaluated for nephrotic syndrome as a routine part of the differential diagnosis workup. Renal biopsy is an essential part of the diagnostic process. This assessment may have to be adapted in the future with the evolution of new antibodies and other non-invasive diagnostic options (e.g., PLA2R-ab and Nephrin-Ab). General management involves the investigation of the underlying disease, identifying complications, and managing the symptoms of the disease.

The pathophysiology of edema formation in nephrotic syndrome has been comprehensively discussed elsewhere as well as in a recent issue of this journal [2]. The role of hypoalbuminemia in edema formation is probably overestimated. Both the plasma and interstitial oncotic pressures in patients with nephrotic syndrome are often reduced and therefore do not significantly contribute to edema formation [3]. The abnormal activation of the epithelial sodium channel in the collecting duct is a key factor in the pathophysiology of edema formation and renders specific therapeutic implications [4,5].

Furthermore, disease entity seems to play an important role, and the same levels of proteinuria and hypalbuminemia may have different clinical phenotypes in different underlying diseases also with regard to edema formation and thromboembolic risks. The risk of thromboembolism is increased in all entities of nephrotic syndrome but is most pronounced and established in patients with membranous nephropathy, with more than 1/3 of all patients affected [6]. The increased risk for thromboembolism in patients with nephrotic syndrome is triggered by hemostatic derangements through increased urinary losses of antithrombotic factors and increased hepatic production of prothrombotic factors as well as increased platelet activation and decreased fibrinolytic activity [6,7]. Localized clotting activation in the kidney may also increase the risk for renal vein thrombosis. Prophylactic anticoagulation is sometimes indicated depending on the underlying disease, the extent of the hypoalbuminemia, proteinuria, and comorbidities [8].

Dyslipidemia is a complication that practically always occurs in persistent nephrotic syndromes [9]. Prolonged dyslipidemia in nephrotic syndrome is associated with an increased risk of cardiovascular events. This association is confounded by other contributing factors such as diminished kidney function, hypertension, and duration of disease [10]. Prolonged duration of disease and failure to achieve remission are associated with various complications, which may result from the ongoing nephrotic syndrome but also often from the exposure to toxic medication used to induce remission.

In this work, we aim to present a comprehensive review of all aspects of entities that are associated with nephrotic syndromes with updated information on recent advances in each field. This includes podocytopathies (focal segmental glomerulosclerosis and minimal-change disease), membranous nephropathy, membranoproliferative glomerulonephritis, IgA nephropathy, fibrillary glomerulonephritis, amyloidosis, and monoclonal gammopathy of renal significance in the context of the nephrotic syndrome. We also elaborate on renal involvement in systemic diseases, diabetic nephropathy, and drugs that are associated with nephrotic syndromes.

## 2. Diseases Associated with Nephrotic Syndromes

### 2.1. Membranous Nephropathy

Membranous nephropathy (MN) is the most common cause of primary nephrotic syndrome in adults. It is defined by a pattern of injury found in the kidney biopsy, showing (a) a thickening of the glomerular basement membrane (GBM) and (b) subepithelial immunoglobulin (Ig)-containing deposits without or with only marginal cell proliferation or infiltration.

### 2.2. MN Classification and Pathophysiology

MN is traditionally categorized based on the suspected etiology into secondary MN, associated with underlying conditions such as systemic autoimmune diseases, infections, or malignancies, and primary (idiopathic) MN, traditionally defined as the absence of such underlying conditions.

Seminal work by Beck et al. identified auto-antibodies targeting phospholipase A2 receptor (PLA2R) on podocytes to be present in patients with primary MN [11]. In 2014 thrombospondin type-1 domain containing 7A (THSD7A), a second podocyte antigen, was discovered in PLAR2-negative MN patients [2,12]. Subsequent studies estimated, that about 60% of MN patients are positive for anti-PLAR2-antibodies, whereas less than 10% are positive for anti-THSD7A-antibodies. More importantly, a pathogenic effect of these antibodies on podocytes was demonstrated for both anti-PLAR2-ab and anti-THSD7A-ab, classifying MN as an autoimmune disease [13,14]. Since then, numerous additional and potentially pathogenic autoantigens have been described in patients with MN, resolving in a classification that better reflects the evolving understanding of the underlying pathogenesis. Primary MN is now seen as the form of MN in which antibodies form immune complexes with endogenous (podocyte) antigens in the glomeruli. In secondary MN the immune complexes consist of a foreign antigen or a neo-epitope in the subepithelial space serving as a target for antibodies [15].

Pathophysiologically, MN is characterized by immune complexes accumulating in the subepithelial space of the glomerular capillary wall along the GBM. The formation of immune complexes is either caused by the presence of autoantibodies against specific podocyte antigens such as PLA2R and THSD7A or by circulating antigens from the underlying condition in secondary MN. The antigens involved in secondary MN are diverse and may be, depending on the underlying condition, viral antigens in hepatitis B-associated MN or tumor antigens in cancer-associated MN. The immune complexes trigger an inflammatory response through the activation of the classical complement pathway (C-CP) as well as directly inducing podocyte injury, leading to conformational changes, effacement of podocyte foot processes, and eventually apoptosis and detachment from the GBM. The damage leads to increased permeability to proteins, resulting in nephrotic syndrome [16]. As the disease progresses, the continuous deposition of immune complexes results in thickening of the GBM. This may present as “spike formations” on capillary walls in histological examination [17]. In secondary MN caused by viral infections or systemic lupus erythematodes (SLE), subendothelial deposits may appear. In SLE or cancer associated secondary MN, endocapillary hypercellularity or mesangial proliferation may be seen [18].

### 2.3. MN Diagnosis

Clinically, approximately 80% of patients present with nephrotic syndrome. Due to the gradual accumulation of deposits of immune complexes, clinical signs of nephrotic syndrome such as progressive edema and weight gain develop slowly, making it difficult to identify the onset and often allowing a long undetected progression of symptoms. Proteinuria is variable, ranging from subnephrotic to highly nephrotic. Hematuria and glucosuria are also common. Renal function is usually preserved, and blood pressure is normal in most patients on presentation.

Kidney biopsy is the gold standard for diagnosis of MN, but a validated serologic-based diagnostic approach has recently emerged [19]. Patients with suspected MN, e.g., nephrotic syndrome or unexplained proteinuria should be evaluated for secondary causes, like infections, autoimmune diseases, medication history or malignancies, and monoclonal gammopathy. All patients should be tested for PLA2R and THSD7A autoantibodies. If anti-PLA2R-ab serology is positive with no evidence of secondary causes and kidney function is normal, the diagnosis of primary PLA2R-associated MN can be made without a kidney biopsy [20]. It is unclear whether this also applies to THSD7A-positive patients. There is a paucity of data in patients with impaired kidney function or evidence of secondary causes of MN, a kidney biopsy should be performed to exclude secondary causes or superimposed diseases. Patients with negative serum PLA2R testing should also undergo kidney biopsy, including examination for PLA2R-staining if findings are consistent with MN. If staining for PLA2R is negative, subsequent testing for further antigens should be performed (Table 1) [21].

### 2.4. MN Treatment

All patients with MN should receive antiproteinuric and nephroprotective therapy with ACE-i/ARB and SGLT2 inhibitors [22,23]. In addition, cardiovascular risk factors should be reduced through a low salt diet, lowering blood pressure (<120 mmHg systolic), and reducing hyperlipidemia. Initial immunosuppressive therapy is restricted to patients with moderate to high risk for progressive kidney injury (Table 2). Patients with proteinuria <3.5 g/d, serum albumin >3 g/dL, and normal kidney function are considered at low risk for kidney injury and may be monitored closely for 3–6 months without immunosuppression. Similarly, patients with proteinuria >3.5 g/d and normal kidney function should receive ACE-i/ARB and SGLT2i to reduce proteinuria by at least 50% within 6 months. If no reduction in proteinuria is achieved, immunosuppressive therapy should be initiated. Recommendations on the choice of immunosuppressive agents and regimens are based on four important landmark trials in primary membranous nephropathy (Table 3) and recent KDIGO guidelines [24]. Patients at high or very high risk should be initiated on immunosuppressants immediately to prevent further loss of function. Life-threatening nephrotic syndrome or rapidly deteriorating kidney function should be treated with a combination of cyclophosphamide and glucocorticoids. For patients with moderate risk, rituximab is the therapy of choice as it has proven efficacious with fewer side effects than alkylating agents. Alternatively, calcineurin inhibitors may be used in moderate-risk patients. For patients classified at high risk of progressing to end-stage kidney disease (ESKD), cyclophosphamide and glucocorticoids remain the main therapy.

Anti-PLA2R-ab concentration may be used to monitor therapy response as a decline usually precedes a reduction in proteinuria [20]. Undetectable anti-PLAR2 titers may allow for discontinuation of immunosuppressive therapy whereas an increase in titers calls for modification of therapy.

### 2.5. New Treatment Strategies

The new generation anti-CD20-antibody obinutuzumab has proven more efficient in depleting CD20-positive B cells and might be efficacious also in patients who did not respond to rituximab [29]. Similarly, the depletion of plasma cells using bortezomib or daratumumab may be considered in refractory cases [30,31]. With the development of cell-specific approaches, future therapies may target the antibody-producing B cell/plasma cell clones alone, avoiding the risk of general immunosuppression. Chimeric autoantibody receptor (CAR) T cells express an epitope of the antigen (i.e., PLA2R) and thus bind to and eliminate specifically B cells that express the respective B cell receptor (i.e., anti-PLA2R), leaving the rest of the immune system untouched [32]. Although still in preclinical development, these cell-specific approaches will likely revolutionize future therapies in MN and beyond.

## 3. Renal Manifestations of Systemic Autoimmune Diseases

### 3.1. Nephrotic Syndrome in Systemic Autoimmune Disease

A variety of different systemic autoimmune diseases may cause or trigger nephrotic syndrome with systemic lupus erythematodes (SLE) being the most prevalent one.

Lupus nephritis (LN) is one of the most common and severe organ manifestations of SLE, with up to one-third of patients developing nephritis in the course of the disease and about 10% progressing to ESKD [33]. Younger age, male sex, and African descent or Hispanic ethnicity as well as Apol1 G1 and G2 risk alleles are associated with worse renal outcomes [34]. In addition, patients with frequent relapses and proteinuria above 4g/day are at higher risk of progressing to ESKD.

### 3.2. Pathogenesis of Membranous LN

The pathogenesis of membranous LN is complex and has been recently reviewed in depth [35]. It involves incomplete clearance of nuclei from apoptotic cells which, in combination with abnormal B- and T- lymphocyte activation, leads to autoantibody formation against nuclear antigens (antinuclear antibodies, ANA) including double-stranded DNA (anti-dsDNA-ab) and antibodies against C1q, Sm, Ro, ubiquitinin, laminin, chromatin, ribosomes and others. Activation of neutrophils and dendritic cells as well as high interferon alpha levels are other hallmarks of LN.

In membranous LN (also termed class V LN), cationic antigens capable of permeating the anionic glomerular basement membrane (GBM) may deposit in the subepithelial space with subsequent antibody binding and immune complex formation in situ. In addition, circulating antibodies may be directed against podocyte-specific antigens, some of which have been identified in recent years including exostosin 1/exostosin 2 complex (EXT1/2), neural cell adhesion molecule 1 (NCAM) and transforming growth factor beta receptor 3 (TGFBR3) [36,37,38]. Both mechanisms may result in local complement activation but no immune cell attraction because the chemoattractant is separated from the blood by the GBM. The injury is, thus, limited to the podocyte, resulting in nephrotic range proteinuria without a decrease in kidney function in most cases.

### 3.3. Diagnostic Workup

While proteinuria > 500 mg/day, active sediment or a decline in kidney function in SLE patients is highly suggestive of LN, a diagnostical kidney biopsy remains the gold standard. Kidney involvement is often underestimated because the renal phenotype is often seemingly mild even in proliferative lupus nephritis (e.g., LN class III and IV). The majority of LN patients (84%) in the phase III trial of belimumab in LN (BLISS-LN) had LN class III or IV, but the mean eGFR in this cohort was completely preserved with 100.5 mL/min/1.73 m^2^ [39]. Several studies also demonstrated low levels of proteinuria even in severe LN, including patients with class III and/or IV LN patients with proteinuria as low as <0.25 g/g creatinine [40,41].

Depending on the extent and site of immune complex deposition, LN is classified into six histopathological groups according to the ISN/RPS classification [42]. Membranous LN is present in up to 20% of LN patients and may occur in combination with class III or IV LN (Table 4) [43]. Pure class V LN shows thickening of the glomerular capillary wall on light microscopy and subepithelial immune deposits on electron microscopy, findings similar to membranous nephropathy (MN). However, while a positive staining of IgA, IgM, IgG, C3, and C1q along more than half of the glomerular capillary loops on immune fluorescence and some mesangial deposits on EM is typically observed in membranous LN, these findings are not seen in MN. Similarly, up to 70% of primary MN show positive IF stainings for PLA2R, which rarely occurs in membranous LN, and the deposited IgG subclass is predominantly IgG4. The serological workup includes the detection of autoantibodies as well as complement factors, and a viral screening.

### 3.4. Treatment

Treatment of membranous LN aims at reducing proteinuria < 0.5 g/day within 18–24 months (complete clinical response) or at least a 50% reduction within 6–18 months (partial clinical response), prevention of decline in kidney function and resolution of serological and extrarenal activity [44]. Normalization of complement levels and >25% reduction in proteinuria after 8 weeks of treatment suggests a favorable kidney outcome [45]. As with every other renal manifestation of SLE, patients with membranous LN should receive anti-inflammatory treatment with hydroxychloroquine in addition to general supportive treatment (i.e., RAAS inhibitor, SGLT-2 inhibitor, salt restriction, anticoagulation if indicated). If the histopathological findings suggest concurrent LN class III or IV, the treatment approach follows the recommendation for LN class III/IV alone [44].

Pure class V LN with nephrotic syndrome or decline in renal function warrants immunosuppressive treatment. In addition, proteinuria > 1 g/day, despite optimal supportive treatment for at least 3 months, warrants treatment since, in contrast to membranous nephropathy, membranous LN shows less tendency for spontaneous remission, and the amount of proteinuria increases the risk of progression to ESKD. The choice of agent remains controversial as recommendations are based on small RCTs or post hoc analyses of larger trials. In addition, ethical susceptibility and economic factors have led to divergent recommendations between the European and North American guidelines and the guidelines issued by the APLAR (Asian-Pacific League of Associations for Rheumatology) [46]. In general, immunosuppressive treatment consists of glucocorticoids with either mycophenolate, cyclophosphamide, or a calcineurin inhibitor (CNI), with MMF being the primary drug of choice. CNIs may be added to glucocorticoids and MMF in severe forms of membranous LN, taking advantage of their stabilizing effect on podocyte cytoskeleton [47]. Because of potential nephrotoxicity, current guidelines recommend against them if the kidney function is severely impaired (i.e., GFR < 45 mL/min). In our experience, however, CNI therapy appears safe even at low or progressively declining rates of eGFR if drug levels are monitored regularly [48,49].

The induction therapy is followed by maintenance therapy with MMF or azathioprine if a complete remission was achieved. Its duration remains controversial, but therapy should be continued for at least 36 months. The appearance of a nephritic sediment should trigger a repeat biopsy to detect LN class switches. Recently, an algorithm for immunosuppression withdrawal was proposed, suggesting a repeat biopsy to assess histological activity after 36–48 months of maintenance therapy in clinically stable patients. If no activity is observed, immunosuppression may be discontinued [50].

Recently, voclosporin, a new-generation CNI that does not require drug-level monitoring, was approved for the treatment of LN in combination with MMF and low-dose glucocorticoids [51,52]. But data on the extent of benefit in membranous LN remains scarce. Similarly, therapy with the anti-BAFF-antibody belimumab—while effective in proliferative LN class III/IV—showed fewer clear results in pure class V LN [53]. Whether this observation is due to true ineffectiveness or an inappropriate study design (i.e., no protocol biopsy, overly strict definitions of complete remission) remains elusive. Consequently, while current guidelines recommend belimumab in combination with MMF or low-dose cyclophosphamide for proliferative LN (i.e., class III and IV), no such recommendation is given for patients with class V LN or patients with nephrotic range proteinuria [44]. As with classical MN, it is likely that the discovery of more endogenous antigens will result in a more detailed (sub-)classification informing targeted therapy.

#### Prognostic Markers

It is now well recognized that repeated renal flares lead to irreversible kidney damage and are thus an independent marker for worse renal outcomes. However, predicting renal outcomes at first presentation remains challenging. Proteinuria, haematuria, and serological markers at the time of active disease do not correlate well with long-term kidney outcomes. Similarly, while important for the correct classification of LN (and thus treatment choice), a robust prediction tool regarding renal outcome using the initial kidney biopsy is lacking. However, histopathological workup should include the extent of tubulointerstitial injury as its importance for short- and long-term prognosis has been demonstrated [54,55]. In addition, robust data suggests a decrease in proteinuria within the first 6 month of treatment, and proteinuria < 700 mg/d at month 12 after treatment initiation reflects a good long-term renal outcome [56,57].

Current research focuses on other markers such as immune cells in the urine or peripheral blood mononuclear cells using high throughput methods such as cytometry by time of flight (CyTOF) or multi-omic approaches [58,59]. Similarly, protocols with repeat biopsies are now being tested to evaluate treatment response and associations between chronic tissue damage and long-term outcomes (i.e., REBIOLUP study, NCT04449991).

### 3.5. Other Systemic Autoimmune Diseases

While membranous LN is a well-recognized disease entity, the association between nephrotic syndrome and other systemic immune diseases is less well-defined and based on case studies. In patients with autoimmune thyroiditis who develop renal disease, most cases present with nephrotic syndrome. About 20% show histopathological findings similar to MN [60]. Both in situ immune response against “planted” thyroglobulin (TG) or thyroperoxidase (TPO) in the subepithelial space as well as deposition of circulating immune complexes consisting of TG and anti-TG-antibodies have been implicated in disease formation [61].

In contrast, only anecdotal associations between nephrotic syndrome and other autoimmune diseases have been described.

While renal crisis is a major concern in patients with systemic sclerosis, nephrotic syndrome is scarce, with case reports primarily describing histopathological findings similar to minimal change diseases or FSGS without proven causal link [62,63].

In sarcoidosis, a wide spectrum of glomerular lesions may be found, including secondary MN, MCD, or FSGS [64]. Similarly, both Sjögren’s syndrome and hypocomplementemic urticarial vasculitis syndrome (HUVS) may present with nephrotic syndrome. The histopathological pattern of injury mainly includes membranoproliferative, mesangioproliferative, and membranous glomerulonephritis in HUVS [65], as well as FSGS and IgA nephropathy in Sjögren’s syndrome [66,67].

Although yet to be proven, a causal relation between these systemic autoimmune diseases and the respective renal pathologies seems plausible, as a dysregulated immune system is the common denominator.

## 4. IgA Nephropathy

IgA nephropathy (IgAN) is the most common primary glomerulonephritis globally and presents with a wide spectrum of clinical manifestations, from asymptomatic microscopic hematuria to severe and rapidly progressive renal impairment. It is characterized by the deposition of IgA immunoglobulins in the mesangial region of the glomeruli.

### 4.1. Pathophysiology

**Figure 1:**
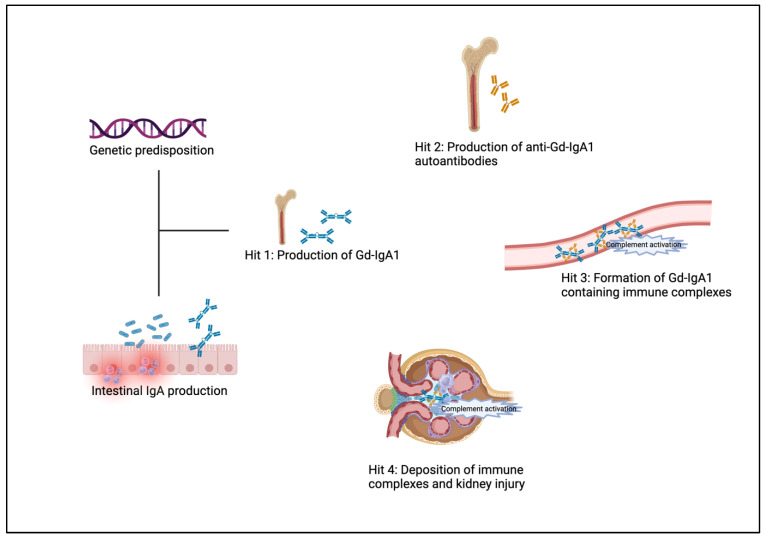
Pathophysiology of IgAN. (Adapted from [78]).

The complex pathogenesis of IgAN is currently understood as a “four-hit” process of immune abnormalities (Figure 1) [68].

The first hit is the production of galactose-deficient immunoglobulin A1 (Gd-IgA1). Patients with IgAN have increased levels of circulating IgA1 with galactose-deficient O-glycans in the hinge region. Gd-IgA1 is thought to originate from cells in mucosal tissues. As an alternative hypothesis, polymeric IgA1 may be produced in the bone marrow due to altered homing of Gd-IgA1-producing plasma cells. The triggers for Gd-IgA1 production are unknown. The B-cell activating factor (BAFF) and a proliferation-inducing ligand (APRIL) have been assumed to play a significant role in the production of Gd-IgA1 since these factors induce a class switch of mucosal B cells into IgA-producing plasma cells. Serum levels of APRIL correlate with disease severity. The second hit describes the formation of IgG- or IgA-autoantibodies to Gd-IgA1, which bind to Gd-IgA1 to form circulating immune complexes (hit 3). Gd-IgA1 containing immune complexes have a predilection for accumulation in the glomerular mesangium. The deposited immune complexes induce kidney injury (hit 4) via activation of inflammatory and cellular proliferative signaling cascades. This leads to activation of mesangial cells, cellular proliferation, and overproduction of extracellular matrix components and cytokines such as interleukin 6 (IL-6) and transforming growth factor β (TGF-β). Mesangial cell activation does not only lead to changes within the mesangium but can also influence the podocyte phenotype via cytokines and other mesangial cell-derived soluble mediators [69].

Complement activation is considered to play an important role in the pathogenesis of IgAN [70,71,72]. Activation commonly occurs locally in glomeruli but can also take place systemically through immune complexes. The alternative pathway has been shown to be the main activator of the complement cascade in IgAN. C3 is co-deposited with Gd-IgA1 in over 90% and correlates with disease progression. Other components and regulators of the alternative pathway, such as complement factor H and complement factor H-related proteins, are found in kidney biopsies and have been shown to be involved in the pathogenesis of IgAN. The lectin pathway has also been shown to be activated in IgAN and to correlate with the severity of the disease. Mannose-binding lectin and C4 are found in deposited immune complexes. The deposition of C4d was found to be associated with worse clinical and histologic characteristics and is an independent risk factor for end-stage kidney disease in IgAN. The terminal pathway also seems to play an important role in the development of IgAN: C5b-9 deposition is associated with kidney inflammation and the progression of glomerulosclerosis. Furthermore, C5b-9 deposition may lead to podocyte damage. Deposition of the different complement components is associated with worse kidney prognosis in IgAN [73].

Activation of the renin-angiotensin system occurs early in the disease process. Endothelin (ET)-1, a growth factor, has been associated with vasoconstriction, mesangial cell proliferation, podocyte damage, production of extracellular matrix, inflammation, and fibrosis and acts via its two receptors (ET-A and ET-B receptor). Increased ET-1 staining was seen in patients with IgAN [74]. The above-mentioned processes lead to glomerular permeability and injury of the podocytes and proximal tubular epithelial cells, resulting in proteinuria and hematuria that frequently progresses to sclerosis, tubular atrophy, and interstitial fibrosis to manifest clinically as decreased renal function.

Susceptibility to IgAN is influenced by genetic and environmental factors. Current genetic research suggests that IgAN is a genetically heterogeneous condition that does not follow classic Mendelian inheritance attributed to a single gene locus. Environmental factors may play a role in the pathogenesis of IgAN. Production of Gd-IgA1 may result in response to a mucosal infection or from a dysregulated mucosal IgA response to food antigens. There is an association between several systemic conditions and the development of histologic and clinical manifestation of IgAN (Table 5). In this context, kidney disease is often referred to as “secondary IgAN”. It is suggested that shared pathophysiologic processes underlie this association [69,75,76,77].

### 4.2. Pathology

Immunofluorescence microscopy reveals characteristic dominant or codominant mesangial IgA staining. This is accompanied by staining of IgG and IgM staining in variable degrees. Glomerular deposition of complement factor C3 shows positive staining in 90%, and C1q is usually negative. The main findings in light microscopy include mesangial hypercellularity and mesangial matrix. In the later course of the disease, segmental or global glomerulosclerosis, tubular atrophy, and interstitial fibrosis are common. Electron microscopy typically shows mesangial electron-dense, immunocomplex deposits. These deposits have also been identified in subendothelial and subepithelial space in up to 30% of cases.

A pathologic classification of IgAN (Oxford Classification/MEST-C Score) was developed by the International IgA Nephropathy Network in collaboration with the Renal Pathology Society. This pathologic scoring system is based upon clinical data and kidney biopsies and scores kidney biopsies upon histologic variables that have been shown to independently predict kidney outcome in IgAN [76,79].

### 4.3. Clinical Features

There is substantial variability in the clinical course of IgAN [24,76]. The most common presentation of IgAN in adults is asymptomatic hematuria with or without proteinuria. Decreases in kidney function can be progressive over the course of the disease. Less common manifestations include macroscopic hematuria (10–15%), nephrotic syndrome, rapid progressive glomerulonephritis (RPGN), and acute kidney injury (AKI). In synpharingitic macroscopic hematuria, patients develop visible hematuria at the same time as an upper respiratory infection or, less commonly, gastrointestinal infection.

Nephrotic syndrome is a rare presentation of IgAN and is discussed separately (see “IgAN as a cause of nephrotic syndrome”). Nephrotic syndrome should be distinguished from the more common presence of nephrotic-range proteinuria (without edema or hypoalbuminemia) in IgAN patients.

### 4.4. IgAN as a Cause of Nephrotic Syndrome

In rare cases, patients with IgAN present with nephrotic syndrome. Typically, there is a correlation between clinical and pathologic findings in patients with IgAN. Patients with mild mesangial proliferation usually present with hematuria, subnephrotic proteinuria, and normal renal function. Findings of more advanced pathologic changes are associated with more severe proteinuria and impaired renal function [80]. In the subset of IgAN patients with nephrotic syndrome, kidney biopsy shows discordant findings of only mild histologic lesions and mesangial deposition of IgA. In these patients, electron microscopy shows extensive foot process effacement, comparable to that seen in minimal change disease (MCD). IgAN with clinical presentation of nephrotic syndrome and features of diffuse foot process effacement on electron microscopy is defined as MCD-IgAN [76].

It is unclear whether this is a specific podocytopathic variant of IgAN or the existence of MCD in a patient with IgAN [80,81,82].

As mentioned above, mesangial cells are activated by deposited Gd-IgA1 containing immune complexes and release inflammatory cytokines. These cytokines not only lead to local changes in the mesangium but can also cause changes in the podocyte phenotype. However, this typically results in subnephrotic range proteinuria [80]. Recent findings suggest a common pathophysiology of Gd-IgA1 deposition in patients with MCD-IgAN and IgAN. Since the clinical course of IgAN-MCD patients was more similar to that of patients with MCD than IgAN, MCD was assumed to be superimposed on indolent IgAN [82]. A recent retrospective study showed that patients with IgAN-MCD had higher levels of proteinuria, lower levels of serum albumin, higher estimated glomerular filtration rate (eGFR), and lower urine blood cells compared to patients with IgAN (excluding MCD-IgAN). Light microscopy showed milder histological lesions and electron microscopy showed weaker fluorescence intensity of IgA in patients with MCD-IgAN. Furthermore, lower levels of Gd-IgA1 and anti-GdIgA1 autoantibodies were detected in MCD-IgAN patients. Purified poly-IgA1 complexes from MCD-IgAN patients induced less inflammatory response in of mesangial cells in vitro. These findings support MCD-IgAN as a dual glomerulopathy consisting of mild IgAN and superimposed MCD [81].

### 4.5. Diagnosis

Diagnosis is confirmed by kidney biopsy, with immunofluorescence demonstrating the presence of deposition of IgA. A kidney biopsy may not be required in every patient with suspected IgAN, depending on the clinical presentation. Since the diagnosis does not alter the course of treatment in patients with isolated hematuria without proteinuria and impaired kidney function, a biopsy is not usually performed in those patients. Indications for kidney biopsies vary geographically.

There are no specific laboratory findings that can be used to diagnose IgAN. Although several serum and urine biomarkers have been tested as diagnostic biomarkers, none of these tests have clearly been shown to have utility. After diagnosis of IgAN, all patients should be assessed for secondary causes [76].

### 4.6. Therapy

Progression to end-stage kidney disease occurs in 25–30% of patients within 20 to 25 years of presentation. The remaining patients enter a sustained clinical remission or have persistent low-grade hematuria and/or proteinuria. Important clinical risk factors include proteinuria, decreased kidney function at presentation, and hypertension. A patient’s risk of progressive disease should be assessed using the International IgAN prediction tool [83]. The tool calculates the five-year risk of a 50% decline of eGFR or progression to ESKD based on clinical and histologic (MEST-C Score) variables at the time of kidney biopsy [76]. This prediction tool is available online at “https://qxmd.com/calculate/calculator_839/international-igan-prediction-tool-post-biopsy-adults (accessed on 26 September 2024)”. While this prediction tool should be used only at the time of biopsy, an updated Prediction Tool can be used for risk stratification one or two years post-biopsy [84]. A urinary peptide classifier (IgAN237) can also predict progressive loss of kidney function in patients with IgAN [85]. Recent data from a UK IgAN cohort presented long-term outcomes that are generally poorer than previously thought, with only a few patients avoiding kidney failure in their lifetime. Even patients traditionally regarded as being low risk, with proteinuria < 0.88 g/g, had high rates of kidney failure within 10 years. We found 30% of patients with time-averaged proteinuria of 0.44 to <0.88 g/g and approximately 20% of patients with time-averaged proteinuria <0.44 g/g developed kidney failure within 10 years [86], which emphasizes the need for optimal supportive care and the implementation of the new therapeutic options developed in recent years.

#### 4.6.1. Supportive Care

Despite advances in understanding the pathophysiology of IgAN, there is, to date, no disease-specific treatment. Supportive care is particularly important in the management of all patients with IgAN and is the first-line treatment in the absence of a rapidly progressive decline in kidney function. Table 6 shows an overview of supportive care.

In the large subgroup of patients with IgAN in the phase 3 DAPA-CKD trial, dapagliflozin showed a remarkable benefit in slowing the progression of chronic kidney disease. Dapagliflozin also reduced the urine albumin-to-creatinine ratio by 26 percent relative to placebo [87]. The primary outcome (a composite of sustained decline in eGFR of at least 50%, end-stage kidney disease, or kidney-related or cardiovascular death) was relatively reduced by 71% (HR 0.29) [88]. The results of the EMPA-KIDNEY trial showed a lower risk of progression to kidney disease or death from cardiovascular events in patients with empagliflozin therapy compared to placebo. This was shown in a broad range of CKD patients, including many patients with IgAN. The risk of progression to end-stage kidney disease sustained eGFR of less than 10 mL/min/1.73 m^2^ or death from kidney failure was reduced by 31%, with similar effects across all categories of kidney disease. The relative reduction in the chronic rate of eGFR decline was −43% in IgAN patients [89].

Several trials focus on the role of new agents for kidney and cardiovascular protection in proteinuric chronic kidney disease. In the phase 3 PROTECT study, sparsentan, a dual-acting angiotensin II and endothelin type A receptor antagonist, demonstrated a significant reduction in proteinuria and preservation of kidney function [90]. Thus, sparsentan has recently been approved for the treatment of IgAN patients with a UPCR of ≥0.75 g/g.

#### 4.6.2. Immunosuppressive Therapy

Immunosuppressive therapy should be reserved for patients who remain at high risk for progression to end-stage kidney disease despite maximal supportive care. The KDIGO 2021 Clinical Practice Guideline for the Management of Glomerular Diseases [24] suggests a 6-month course of glucocorticoid therapy in those patients. The important risk of treatment-emergent toxicity must be discussed with patients, particularly in those who have an eGFR < 50 mL/min/1.73 m^2^. If patients have evidence of severe and irreversible kidney damage (eGFR < 30 mL/min/1.73 m^2^ for >3 months, small echogenic kidneys on kidney ultrasound, or evidence of severe interstitial fibrosis, tubular atrophy, or glomerulosclerosis on kidney biopsy), immunosuppressive therapy is unlikely to be effective [24,76].

Corticosteroids are the only currently available immunosuppressive agent with supporting, though conflicting, evidence as therapy in IgAN. Recent trials have questioned the benefits for most patients.

The STOP-IgAN study showed that the addition of immunosuppressive therapy to supportive care was superior to supportive care alone in inducing remission of proteinuria; however, there was no significant effect of immunosuppressive therapy on eGFR. Immunosuppressive therapy was associated with higher rates of adverse events [91].

Another trial, the TESTING study, showed a significant reduction in the primary composite endpoint (40% decline in eGFR, development of kidney failure, death from kidney disease) compared to placebo. However, the treatment was associated with a higher incidence of serious adverse events, particularly infections, which raised concerns about the safety of long-term steroid use in these patients [92]. The trajectory of renal function decline in the control group was four times faster in the TESTING trial compared to that in the STOP-IgAN trial, suggesting a higher-risk (mainly Asian) study population and/or disparities in supportive therapy, which may explain the different findings. In the selection of corticosteroids for the treatment of IgAN, renal benefits must be weighed against the risk of serious adverse events.

#### 4.6.3. New Forms of Immunosuppressive Therapy

**Figure 2:**
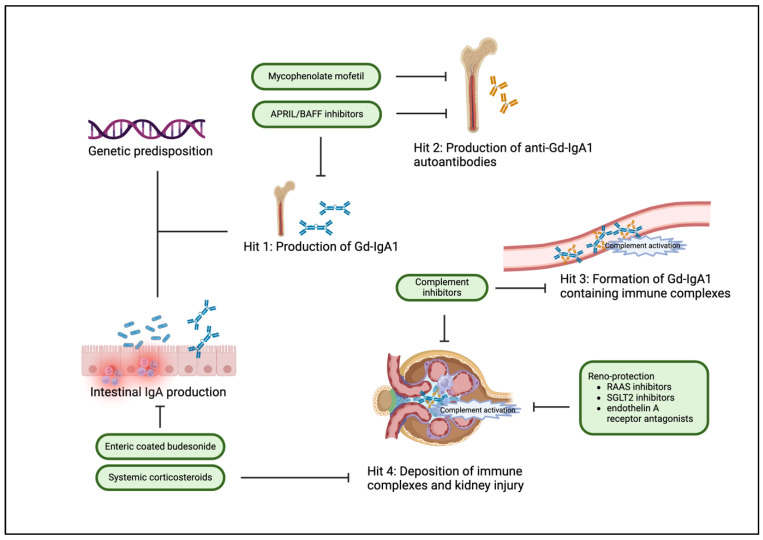
Pathophysiology of IgAN targeted by current and new drugs (adapted from [78]).

For patients who are unable to tolerate or do not wish to take systemic glucocorticoids, the targeted-release formula (TRF) of budesonide may be an alternative option for initial immunosuppressive therapy. The rationale for TRF-budesonide was to release the drug at the distal ileum, where the largest site of Gd-IgA1 secreting cells is located, the mucosal-associated lymphoid tissue (Figure 2). A subsequent reduced production of Gd-IgA1 is hypothesized. A phase 3 study showed that TRF-budesonide significantly reduced proteinuria and slowed the decline in kidney function compared to placebo [93].

Data in Asian populations showed proteinuria reductions with the use of MMF. Recent data demonstrated its use as a steroid-sparing agent. A regimen combining mycophenolate mofetil with low-dose prednisone for 6 months was found to be as effective as full-dose prednisone in achieving proteinuria remission at 6 and 12 months, with fewer adverse events [94]. However, there is a lack of sufficient data to confirm the efficacy of mycophenolate in other populations, particularly in whites or in patients with advanced stages of the disease [76]. The KDIGO 2021 guideline suggests not using mycophenolate as a treatment for IgAN [24].

Interesting results from China pointed to a potentially disease-modifying effect of hydroxychloroquine (HCQ). In addition to RAAS inhibition, HCQ significantly reduced proteinuria in patients with IgAN over 6 months without evidence of adverse events [95].

In addition to systemic and local glucocorticoids, other immunosuppressive agents such as calcineurin inhibitors, rituximab, and cytotoxic agents have been assessed for the treatment of IgAN. Given the lack of clear evidence supporting their efficacy in patients with IgAN, they are not routinely recommended or used.

#### 4.6.4. Investigational Agents

Given the recent advances in the understanding of the pathogenesis of IgAN, a number of novel investigational agents for the therapy of IgAN are being evaluated in clinical trials (Table 7).

As mentioned above, activation of the alternative and lectin complement pathway plays a key role in the pathogenesis of IgAN, and several complement inhibitors are being investigated in current trials.

MASP-2 is the effector enzyme of the lectin pathway. The efficacy of narsoplimab, an inhibitor of MASP-2, was evaluated in a recent phase 3 study (ARTEMIS-IGAN trial). The trial was discontinued since treatment with narsoplimab did not result in a significant reduction in proteinuria compared with a placebo.

The inhibition of the alternative pathway is also being investigated. A phase 2 trial in patients with IgAN showed a dose-dependent reduction in proteinuria with the factor B inhibitor iptacopan compared to placebo [96]. A phase 3 trial is in progress.

Selective inhibition of C3 is also being tested in patients with IgAN. The efficacy of pegcetacoplan, a factor C3 inhibitor, in reducing proteinuria is being evaluated in a phase 2 study. Furthermore, different inhibitors of C5, such as ravulizumab and cemdisiran, that target the common complement pathway are being tested in current trials.

BAFF and APRIL are critical in IgA class switch recombination and thus in the production of Gd-IgA1. The efficacy of BAFF and APRIL inhibitors is being evaluated in clinical trials. In a phase 2 study, sibeprenlimab, an APRIL inhibitor, was shown to lead to a greater reduction in proteinuria compared to a placebo in patients with IgAN [97]. A phase 3 trial is in progress. Additionally, other BAFF/APRIL inhibitors such as povetacicept, atacicept, and telitacicept are being investigated in patients with IgAN.

Atrasentan, an endothelin A receptor inhibitor, is being investigated in addition to the above-mentioned and already approved sparsentan.

#### 4.6.5. Therapy of Variant and Secondary Forms

Patients with MCD-IgAN should be treated in accordance with the recommendations and guidelines for MCD. Patients with RPGN in IgAN should—despite the lack of well-supporting data—be treated with cyclophosphamide and glucocorticoids in accordance with the guidelines for ANCA-associated vasculitis. In patients with AKI, immediate management should focus on supportive care for AKI. A repeat kidney biopsy should be considered when clear improvement of kidney function does not occur within one week to exclude the possibility of crescentic disease. The optimal treatment approach for secondary IgAN is not well established. Therapy should be directed at the underlying primary disease.

### 4.7. Summary

IgA nephropathy is the most common glomerular disease and a significant contributor to kidney failure. Advances in understanding the molecular mechanisms underlying its pathogenesis could enable earlier diagnosis, enhance monitoring of the disease’s progression or response to treatments, and ultimately lead to the development of targeted therapies.

## 5. Membranoproliferative Glomerulonephritis

The term membranoproliferative glomerulonephritis (MPGN) groups different pathologies, resulting in a specific pattern of glomerular histologic lesions. The characteristic picture of glomerular injury in kidney biopsy is composed of (a) thickening of the glomerular basement membrane (GBM) and (b) mesangial and endocapillary hypercellularity.

### 5.1. MPGN Classification and Pathophysiology

MPGN is classified into three main categories based on immunofluorescence microscopy findings and categorized based on the underlying pathophysiological process. The three categories are the immunoglobulin (Ig)-/immune complex-mediated MPGN (I-MPGN), the complement-mediated MPGN (C-MPGN), and the least frequent MPGN without Ig or complement deposition [98].

I-MPGN is caused by antigenemia and/or circulating immune complexes, as can be seen in chronic infections and autoimmune diseases [99]. It is characterized by predominant immune complex deposits in the mesangial, subendothelial, and subepithelial space, causing an activation of the classical complement pathway (C-CP) and an influx of inflammatory cells. This results in first proliferative and following reparative glomerular changes such as endothelial and mesangial hypercellularity. Immune complexes are trapped by the resynthesis of the GBM, forming characteristic double membrane contours, also known as “tram tracking” [100].

In primary I-MPGN, no causal etiology can be found, whereas in secondary I-MPGN, an underlying systemic disease or infection can be identified (Table 1) [101].

C-MPGN is caused by dominant (defined as >2 fold more intense C3 staining compared to other immune reactants in immunofluorescence) or solitary C3 (or C4) deposits in kidney biopsy. It is subdivided by electron microscopy findings into C3 glomerulonephritis (C3GN), C4GN, dense deposit disease (DDD), and C4-DDD. DDD is characterized by intramembranous sausage- or ribbon-shaped electron-dense deposits in the GBM, whilst C3GN shows more heterogeneous and lighter mesangial, subendothelial, or subepithelial deposits [102].

C3GN results from dysregulation and persistent activation of the alternative complement pathway (A-CP), whereas very rarely, C4GN may result from an overactive lectin pathway [103]. The excessive local activation of the A-CP leads to activation and deposition of C3 breakdown products, triggering inflammation and functional impairment and activation of the terminal pathway with the production of the cytotoxic C5b-9-complex [104]. Abnormal regulation or activation of C3- and/or C5-convertase is caused by either autoantibodies or genetic variants in complement coding genes, leading to a failure of inhibitory proteins. In contrast to atypical hemolytic uremic syndrome, the major site of the uncontrolled excessive A-CP activity is not in the glomeruli but in the fluid phase [105].

### 5.2. MPGN Diagnosis

**Figure 3:**
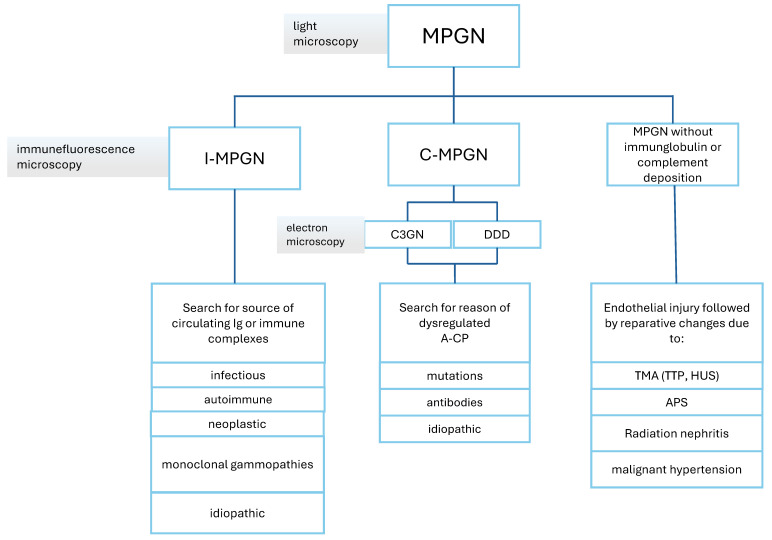
MPGN classification, adapted from [106,107] A-CP, alternative complement pathway; C-MPGN, complement-mediated MPGN; C3GN, C3 glomerulonephritis; DDD, dense deposit disease; HUS, hemolytic uremic syndrome; I-MPGN, immunoglobulin (Ig)-/immune complex-mediated MPGN; MPGN, membranoproliferative glomerulonephritis; TMA, thrombotic microangiopathy.

The clinical presentation of the diagnosis of MPGN is similar to other types of glomerulonephritis. Typically, patients present with hematuria and/or a variable degree of proteinuria. Serum creatinine may be normal or elevated. Some patients report non-specific complaints, such as joint pain and fatigue. In I-MPGN, symptoms of the underlying condition might be found, whereas in DDD, drusen formations may be seen in the funduscopic examination. C-MPGN leads to nephrotic syndrome in 50% of cases. Kidney biopsy is essential for diagnosing MPGN. Subclassification is made based on immunofluorescence microscopy findings [106] (Figure 3).

Regardless of subtype, hypocomplementemia is common but not mandatory for the diagnosis of MPGN. C3 tends to be decreased in C-MPGN, whereas in I-MPGN, low C4 often occurs due to the activation of C-CP.

Upon diagnosis of I-MPGN, secondary forms need to be excluded through an extensive search for triggering underlying diseases. This includes diagnostic evaluation of infectious diseases such as hepatitis B (HBV), C (HCV), and HIV, autoimmune diseases such as systemic lupus erythematosus and Sjögren’s syndrome, and monoclonal gammopathies and neoplasms. (Table 8) It is questionable whether the entity “primary MPGN” exists at all, and in the event of an unsuccessful search for a trigger, a complement analysis is recommended.

Once a diagnosis of C-MPGN has been made, further diagnostic investigations are required to identify the trigger for the uncontrolled activation of the A-CP (Table 9). Autoantibodies can be detected in 50–80% of patients with C-MPGN [108]. The most common are C3 nephritis factor (NeF) and C5NeF, leading to a stabilization of C3 and/or C5-convertase [109,110]. Less common are autoantibodies against the inhibitory proteins complement factor (CF) H and CFB or opsonizing components like C3b [111,112]. 20–25% of patients with C3GN carry genetic variants in complement regulating protein-coding genes. Common forms are mutations in Complement factor H-related (CFRH) genes, such as CFRH1-5. Hereditary forms are CFHR5 nephropathy and CFRH1-3 hybrid genes (Table 9).

### 5.3. MPGN Treatment

Supportive strategies, such as dietary sodium and protein restriction, blood pressure control, RAAS inhibition, sodium–glucose cotransporter 2 (SGLT2) inhibitors, and the avoidance of nephrotoxic substances, are general measures for all patients with MPGN. The treatment depends upon the classification and the identification of the underlying cause and pathogenesis.

In patients with I-MPGN, an underlying cause, such as a chronic infection, an autoimmune disease, or a monoclonal gammopathy, can be found in the majority of cases. These patients should receive therapy against the primary disease. This may include anti-infective therapy, immunosuppression, and plasma cell or B-cell targeting therapy. After a comprehensive workup with the exclusion of I-MPGN and C-MPGN, the rare case of primary-MPGN or better MPGN with the unidentified trigger is leaving the treating physicians with difficult treatment decisions without supporting data from trials.

Patients presenting with mild disease (non-nephrotic-range proteinuria, no hematuria, and normal kidney function) may be treated with supportive therapy only. These patients tend to have a good long-term outcome, and the use of steroids has not been shown to be beneficial [113]. Patients with impaired kidney function, active urinary sediment, and/or nephrotic syndrome should receive immunosuppressive therapy with either glucocorticoids, calcineurin inhibitors (CNIs), or mycophenolate mofetil (MMF), or combinations of these for 3–12 months. In the case of ongoing active GN, cyclophosphamide and rituximab can be reasonable alternative treatment options, although there is a lack of data demonstrating a benefit.

All patients should be closely monitored (serum creatinine and urine albumin excretion). In case of worsening proteinuria, hematuria, or deteriorating kidney function, a re-kidney biopsy should be performed in order to identify the ongoing process and/or re-classify MPGN.

In patients with C-MPGN and monoclonal gammopathy, treatment is primarily directed against the pathologic clone (see MGRS treatment). In the absence of monoclonal gammopathy, C-MPGN is treated depending on the severity of symptoms. A patient presenting with mild disease (proteinuria < 1.5 g/day), no hematuria, and normal kidney function) may be treated with supportive therapy only.

For all patients with moderate to severe disease, initial therapy with MMF and oral glucocorticoids is recommended. In refractory disease with factor H mutation, plasma infusion or plasma exchange could be considered based on a case report [114], and even in patients without proven genetic defects, therapy with eculizumab has been shown to be effective in some patients [115]. Patients who have C-MPGN and rapidly progressive glomerulonephritis (RPGN) should be treated with glucocorticoids and either cyclophosphamide or MMF. A case series suggests the additional administration of eculizumab [116]. Investigational approaches with novel complement inhibitors for the treatment of C-MPGN, such as iptacopan (NCT05755386), Danicopan (NCT03124368), and Pegcetacoplan (NCT05067127), are currently under investigation [117].

### 5.4. What’s New?

The establishment of the new pathogenesis-oriented classification of MPGN (Figure 1) led to a better understanding of MPGN. Recent data showed that apolipoprotein E and complement proteins of the terminal pathway accumulated in DDD deposits in high concentrations compared to less dense deposits in C3GN and ApoE staining may become an adjunct to electron microscopy for the diagnosis of DDD [118]. Different agents targeting either the A-CP or preventing C3 activation, as the converging part of all three pathways, are being tested in phase II and III trials and will likely expand the therapeutic potential for C3G and I-MPGN in the future.

## 6. Podocytopathies: Focal Segmental Glomerulosclerosis and Minimal-Change Disease

Minimal change disease (MCD) and Focal segmental glomerulosclerosis (FSGS) are podocytopathies [119] that commonly result in nephrotic syndrome. MCD accounts for approximately 90% of nephrotic syndrome cases in children. In adults, MCD is less common and accounts for only about 10% of nephrotic syndrome cases. The pathologic hallmark of MCD is the absence of visible alterations by light microscopy and the effacement of foot processes by electron microscopy. Most cases are idiopathic without an identifiable cause or association with an underlying disease. FSGS refers to a pattern of kidney damage that predominantly affects the podocytes. It is characterized by sclerosis seen in parts (segmental) of some (focal) glomeruli under light microscopy. FSGS can be categorized into primary, secondary, and genetic forms based on their underlying causes.

### 6.1. Pathophysiology

In spite of decades of research, the underlying causes of both diseases remain incompletely understood.

#### 6.1.1. Minimal Change Disease

In MCD, a systemic process is thought to lead to the production of a glomerular permeability factor. This circulating factor affects the glomerular capillary wall, causing effacement of the foot processes and leading to proteinuria. Just recently, one factor may have been found with the discovery of autoantibodies against nephrin, a component of the slit diaphragm, in a subset of patients with MCD (see the What’s New? Section below) [120]. However, MCD can be associated with secondary causes such as drugs, malignancies, and infections (Table 10) [121].

#### 6.1.2. Focal Segmental Glomerulosclerosis

Pathogenesis of primary FSGS likely involves a circulating factor that causes generalized podocyte dysfunction manifested as foot process effacement [122]. The precise identity of the factors remains unknown. Some of the most commonly discussed potential circulating factors include cardiotrophin-like cytokine factor 1, serum urine–type plasminogen activator receptor (suPAR), microRNA, and other factors. Damage to the podocyte is the first event in the pathogenic process.

Secondary FSGS can occur as a reaction to direct toxic injury to podocytes, which can be caused by a variety of conditions, such as drugs or viruses. Other forms of secondary FSGS result from adaptive overfiltration in the remaining glomeruli after a reduction in the number of functioning nephrons (Table 11).

Additionally, various genetic forms of FSGS have been identified [123], involving genes that typically code for proteins crucial for the proper functioning of the glomerular filtration barrier, such as nephrin or podocin. A number of susceptibility genes may confer an increased risk of FSGS. The best known of these polymorphisms is the APOL1 gene, especially among individuals of African descent.

### 6.2. Pathology

#### 6.2.1. Minimal Change Disease

On light microscopy, the glomeruli appear normal, and on immunofluorescence microscopy, there are no complement or immunoglobulin deposits. The characteristic finding on electron microscopy is diffuse effacement of the epithelial foot processes [121].

#### 6.2.2. Focal Segmental Glomerulosclerosis

In FSGS, light microscopy typically reveals lesions characterized by segmental consolidation of capillary loops, with the lumen being obliterated. Immunofluorescence microscopy usually shows an absence of immune deposits. Electron microscopy further details these lesions, showing collapsed capillary loops often containing trapped hyaline material. A characteristic feature of FSGS is the effacement of podocyte foot processes, which is, in the majority of cases, diffuse in primary FSGS and segmental in secondary FSGS. The Columbia classification subdivides the lesion of FSGS by its appearance on light microscopy into collapsing, tip lesion, cellular, perihilar lesion, and not otherwise specified variants [124].

### 6.3. MCD and Primary FSGS—Different Manifestations of the Same Progressive Disease?

There is an ongoing discussion as to whether MCD and primary FSGS fall within the spectrum of the same disease or whether they represent two different disease entities. It has been hypothesized that minimal change disease and primary FSGS are part of the same disease spectrum, where both are associated with circulating permeability factors, but primary FSGS represents a more advanced and often more therapy-resistant phenotype. MCD typically presents with full nephrotic syndrome, complete foot process effacement on electron microscopy, and a complete response to immunosuppression. End-stage kidney disease is rare.

In primary FSGS, response to immunosuppression is only seen in half of the patients, typically as partial remissions. Progressive kidney failure is common in patients with persistent high-grade proteinuria.

In patients with steroid-resistant nephrotic syndrome who have normal appearing glomeruli under light microscopy in initial biopsy but later progress to FSGS, some researchers suggest that the initial diagnosis of FSGS could have been missed because of the focal and segmental nature of FSGS lesions. However, evidence suggests that FSGS lesions might be absent in the early phase of the disease, and finding of FSGS lesions in repeat biopsies shows disease progression. In most cases, FSGS is considered a result of podocyte injury, which typically follows an underlying glomerular disease. It has been proposed that the initial injury to the podocytes is a critical event. The extent of this injury, the vulnerability of the podocytes, and the response to treatment determine whether FSGS lesions will form. MCD and primary FSGS share common underlying causes, and multiple causes of initial podocyte injury exist [125].

### 6.4. Clinical Presentation

#### 6.4.1. Minimal Change Disease

Usually, patients with MCD present with a sudden onset with symptoms of nephrotic syndrome over days to a week, often following an upper respiratory or other systemic infection. Microscopic hematuria is common, and serum creatinine may be modestly elevated at presentation. Acute kidney injury is an infrequent complication [121].

#### 6.4.2. Focal Segmental Glomerulosclerosis

Patients with primary FSGS typically present with an acute onset of severe nephrotic syndrome. Hematuria is common, and an elevated serum creatinine may be seen. Patients with secondary FSGS typically present with slowly increasing proteinuria and kidney function impairment. The proteinuria is often in the non-nephrotic range, serum albumin levels are usually normal, and patients usually do not have peripheral edema, even when proteinuria exceeds > 3.5 g/day. Clinical presentation of patients with genetic FSGS can vary, depending upon the specific genetic mutation involved. Glucocorticoid resistance is a consistent feature among patients with monogenic forms of FSGS, although this may also be seen in patients with primary FSGS [122].

### 6.5. Diagnostic

#### 6.5.1. Minimal Change Disease

To diagnose MCD in adults, a kidney biopsy is necessary. There are currently no validated specific laboratory tests that can distinguish MCD from other types of nephrotic syndrome. Given the high prevalence of MCD in children, pediatricians often make a presumptive diagnosis without a biopsy, restricting this procedure to cases where the nephrotic syndrome is resistant to glucocorticoid treatment. Once MCD is diagnosed, it is important to assess patients for possible secondary causes of the condition [121].

#### 6.5.2. Focal Segmental Glomerulosclerosis

**Figure 4:**
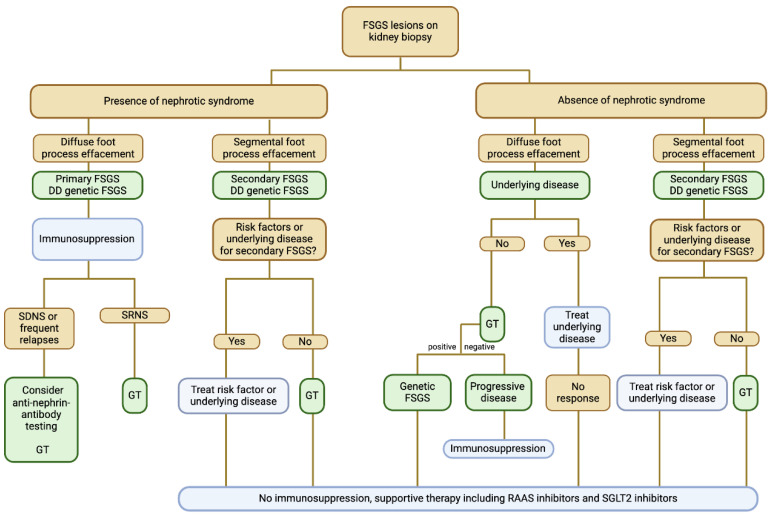
Evaluation of FSGS lesions on kidney biopsy. GT: Genetic testing.

A kidney biopsy is necessary to identify FSGS lesions. As FSGS is a histological pattern rather than a distinct disease entity, the detection of FSGS lesions should lead to an evaluation of potential underlying causes. Distinguishing between primary and secondary FSGS involves assessing the presence or absence of nephrotic syndrome, the degree of podocyte foot process effacement seen on electron microscopy (typically diffuse in primary FSGS and segmental in secondary FSGS) and known risk factors for secondary FSGS (Figure 4). A urine peptide-based classifier showed impressive performance in selectively detecting primary FSGS with high specificity [126].

However, these clinical and pathological features do not identify patients with genetic causes of FSGS. Therefore, genetic testing should be considered in patients who cannot be classified by clinicopathologic assessment. In addition, the presence of a family history of chronic kidney disease or physical signs of syndromic disease should prompt genetic testing. Currently, there are no reliable and clinically useful biomarkers that aid the diagnostic process of classifying FSGS lesions [123].

### 6.6. Therapy

The goal of therapy for patients with MCD and FSGS is remission of proteinuria and preservation of kidney function (Table 12). This is primarily achieved with immunosuppressive agents, most commonly glucocorticoids. Some patients with MCD may experience spontaneous remission without treatment. Nevertheless, immunosuppressive therapy should not be withheld due to the thrombotic, cardiovascular, and infectious risks of persistent nephrotic syndrome. In addition to immunosuppressive therapy, general supportive measures are recommended for all patients with MCD or FSGS. Supportive care encompasses dietary sodium restriction, blood pressure control, renin-angiotensin system inhibition, treatment of dyslipidemia (statins), and, in selected patients, anticoagulation. SGLT-2 inhibition may also be beneficial. Therapy also includes diuretics to treat edema.

#### 6.6.1. Minimal Change Disease

For patients with primary MCD, an initial therapy with glucocorticoid monotherapy is suggested. For patients with contraindications to or who do not wish to take high-dose glucocorticoids, calcineurin inhibitors such as cyclosporine or tacrolimus are an alternative option [127]. Mycophenolate mofetil and cyclophosphamide (with or without low-dose glucocorticoids) have also been applied in small case series. 50–75% of adults will experience a relapse, and glucocorticoid dependence occurs in 25–30%. For patients with infrequent relapses, a repeat course of the initial therapy is suggested. For patients with frequent relapses or glucocorticoid dependence, treatment with cyclophosphamide, rituximab, a calcineurin inhibitor, or mycophenolate mofetil rather than glucocorticoid monotherapy is recommended to avoid the morbidity associated with prolonged glucocorticoid therapy. There is limited evidence directly comparing one drug class with another in the treatment of patients with frequent relapses or glucocorticoid-dependent MCD, and available data have not established that one regimen is superior to another. The therapeutic approach for glucocorticoid-resistant MCD is not known; in general, it is treated with a calcineurin inhibitor with or without low-dose glucocorticoids. Careful re-evaluation of the patient, repeated kidney biopsy, analysis of nephrin-autoantibodies, and genetic testing should be undertaken.

Treatment of patients with secondary MCD focuses on the cessation of the offending drug or effective treatment of the underlying disease in addition to supportive therapy [24].

#### 6.6.2. Focal Segmental Glomerulosclerosis

For patients with likely primary FSGS, initial therapy with glucocorticoids is recommended [24]. Calcineurin inhibitors with or without low-dose glucocorticoids should be considered as the initial therapy in patients who have a high risk for glucocorticoid-induced toxicity. For patients with relapse, a repeat course of prednisone is suggested. If adverse effects occur, or in case of frequent relapses, switching to calcineurin inhibitors with or without low-dose glucocorticoids is preferred. For glucocorticoid-dependent or -resistant FSGS, calcineurin inhibitors are recommended over continued glucocorticoids or no therapy. Glucocorticoid-dependent patients may use calcineurin inhibitors with low-dose prednisone. In case of a lack of response to calcineurin inhibitors, alternative treatment with mycophenolate mofetil or rituximab can be considered in glucocorticoid-dependent patients. There is no high-quality evidence to guide the optimal therapy in glucocorticoid-resistant patients and enrollment in clinical trials should be evaluated. Successful off-label treatment in rituximab-refractory FSGS with obinutuzumab and daratumumab treatment is reported in case reports [128].

Treatment of secondary FSGS focuses on the treatment of the underlying condition or termination of the offending drug. The optimal approach to therapy of genetic forms of FSGS is not known. Patients with secondary and genetic forms of FSGS should receive supportive measures, while treatment with immunosuppressive therapy is, in the majority of cases, not indicated [24].

A deeper understanding of the pathomechanisms underlying FSGS and MCD, particularly regarding new biomarkers like anti-nephrin antibodies, could pave the way for more personalized treatment strategies.

Given the low incidence of MCD and FSGS, particularly in complicated courses, conducting meaningful prospective randomized trials in the adult population is challenging. As a result, there is a greater emphasis on the value of real-world data and clinical registries that might improve our understanding of these diseases.

##### Investigational Therapies in FSGS

Sparsentan, an oral dual antagonist of the angiotensin II and endothelin A receptors, has demonstrated potential in reducing proteinuria in a phase III clinical trial involving patients with FSGS. The trial indicated that a higher proportion of sparsentan-treated patients achieved partial remission compared to those treated with irbesartan. However, sparsentan did not significantly affect the eGFR over 108 weeks [129]. Sparsentan is conditionally approved for treating IgA nephropathy but not for FSGS. Additionally, clinical trials are underway to evaluate the efficacy of atrasentan, another endothelin receptor antagonist, in FSGS.

Inaxaplin (VX-147) is a selective oral inhibitor targeting APOL1 channel function aimed at treating proteinuric kidney disease in FSGS patients with two APOL1 risk alleles. It binds directly to the APOL1 protein, inhibiting channel function and reducing proteinuria, as demonstrated in preclinical mouse models. In a phase 2a study with 16 patients, inaxaplin significantly decreased proteinuria by 47.6% over 13 weeks, with manageable adverse effects [130]. Further studies are required to assess its long-term efficacy and safety.

Other agents under investigation for patients with FSGS include voclosporin, losmapimod (p38 mitogen-activated protein [MAP] kinase inhibitor), PF-06730512 (ROBO2/SLIT2 inhibitor), bleselumab (anti-CD40 monoclonal antibody), abatacept, CCR2 inhibitors, and Nrf2 activators.

### 6.7. What’s New?

In 2022, anti-nephrin autoantibodies were discovered, which are present during active minimal change disease but reduced or absent during treatment response and remission [120]. Hengel et al. recently explored the role of anti-nephrin autoantibodies in various nephrotic syndromes [131]. The study focused on MCD, primary FSGS, and idiopathic nephrotic syndrome in children, aiming to elucidate the prevalence and potential clinical significance of these autoantibodies. Nephrin is a protein of the podocyte slit-diaphragm architecture and has significant signaling functions. Severe podocyte injury occurs on genetic mutation or experimental knockout of nephrin. The study identified that anti-nephrin autoantibodies were present in 44% of adults with MCD and 9% with primary FSGS. Among children with idiopathic nephrotic syndrome, 52% had detectable anti-nephrin antibodies. The levels of these autoantibodies were found to correlate with disease activity. In untreated patients with active MCD or idiopathic nephrotic syndrome, the prevalence of anti-nephrin autoantibodies was 69% and 90%, respectively [131]. A direct pathogenicity and causal role of these autoantibodies in the disease pathogenesis is suggested. It was shown that anti-nephrin autoantibodies induced phosphorylation of nephrin, resulting in profound cytoskeletal alterations. In a few patients with anti-nephrin autoantibodies, therapy with rituximab was shown to deplete the autoantibodies and induce clinical remission. The discovery of anti-nephrin autoantibodies in MCD and FSGS could provide a potential biomarker also for guiding therapy [131].

### 6.8. Summary

MCD and FSGS are podocytopathies associated with nephrotic syndrome. Current diagnostic strategies include kidney biopsy with therapeutic approaches often relying on non-targeted immunosuppressive treatments like corticosteroids and other immunomodulatory drugs, though these treatments are not tailored to the specific underlying causes of the diseases. Current research is focusing on understanding the underlying molecular mechanisms, which could lead to more targeted therapies. In particular, identifying specific biomarkers for early diagnosis and monitoring, as well as developing new treatment strategies that address the root causes rather than just the symptoms, are key areas of interest.

## 7. Amyloidosis and Monoclonal Gammopathy of Renal Significance

Monoclonal gammopathy of renal significance (MGRS) is a clinical entity that is distinct from monoclonal gammopathy of unknown significance (MGUS) and overt multiple myeloma or other lymphoproliferative diseases. In MGRS and MGUS, dysfunctional immunoglobulins (paraproteins), either intact or fragmented, are produced by a dyscratic monoclonal B-cell population. This distinction between MGRS and MGUS was made in 2012 to denote the renal damage caused by the paraprotein and provide a rationale for the treatment of the underlying B-cell dyscrasia while not (yet) meeting the criteria for full hematologic malignancies such as multiple myeloma or CLL. One subtype of MGRS is AL amyloidosis, caused by monoclonal immunoglobulins (fragments) that misfold and aggregate into congophile deposits that cause tissue damage and dysfunction. Outside the context of MGRS, other inherited and acquired forms of amyloidosis may affect the kidneys.

### 7.1. MGRS Classification

Depending on the bio-physical properties, paraproteins in MGRS may be deposited in different renal compartments (interstitial, tubular, glomerular, and vascular) or induce endothelial damage via the complement cascade or vascular endothelial growth factor signaling [132,133,134,135]. In turn, the pathogenic mechanism determines clinical presentation and prognosis (Table 13). While some forms mainly cause acute kidney injury or electrolyte abnormalities, others present as glomerular proteinuria and sometimes even nephrotic syndrome. The most common forms are cast nephropathy, AL amyloidosis, and monoclonal Ig deposition disease (either involving light chains (LCDD, most frequent), heavy chains (HCDD, rare), or light-and-heavy chains (LHCDD, rare)). Of these, AL amyloidosis presents most frequently with heavy proteinuria and nephrotic syndrome, which may also occur in Ig-deposition disease. Rare MGRS entities, such as cryoglobulinemia type 1, crystalline podocytopathy, C3-glomerulopathy, and immunotactoid glomerulopathy, have also been associated with heavy proteinuria and nephrotic syndrome [136].

### 7.2. MGRS Diagnosis

The diagnosis of MGRS should be suspected in the context of acute kidney injury and/or glomerular proteinuria in patients with circulating monoclonal antibodies and confirmed by renal biopsy. Typically, biopsy reveals the site of monoclonal protein deposition, confirms monoclonality using light-chain-restriction analysis, and identifies the mechanism of damage. If AL amyloidosis has been confirmed in a different organ and the patient presents with the typical clinical picture of renal amyloidosis, a renal biopsy becomes obsolete, and the patient can be presumed to have renal involvement in AL amyloidosis.

To confirm the presence of a circulating monoclonal protein, serum immunofixation (identifying the class of paraprotein), serum and urine electrophoresis (allowing quantification of the M-gradient/paraprotein), and a serum-free light-chain assay (to increase sensitivity) should be performed. Of note, monoclonal deposits in the kidney may not be accompanied by a detectable monoclonal protein in serum, either because quantities produced by a small clonal population are below the assay detection limit or because the kidney functions as a sink sequestering all circulating paraproteins quickly. Bone marrow aspirates and biopsies should be performed to rule out myeloma and confirm the presence of a monoclonal plasma cell population with the same restriction pattern as the renal deposits, even if no circulating monoclonal protein can be detected.

Wide differences between total urine protein and urine albumin may indicate the presence of large amounts of monoclonal proteins, but this “proteinuria gap” has been shown to be unreliable in diagnosing multiple myeloma [137] but could be helpful in the diagnosis of myeloma cast nephropathy [138].

### 7.3. MGRS Treatment

Apart from supportive strategies (RAAS inhibition, blood pressure control, anticoagulation, salt restriction, and avoidance of nephrotoxic substances), therapeutic approaches are aimed at targeting the underlying monoclonal cell population. If the criteria for an overt, full hematologic malignancy are met, the best standard of care for hematologic malignancy is advised, irrespective of renal involvement. If the paraprotein originates from a monoclonal plasma cell clone, a plasma cell-directed therapy analog to myeloma protocols is required, and patients should be consulted with a hematologist. Until recently, systematic, randomized trials on MGRS were lacking. In the case of AL amyloidosis, the ANDROMEDA trial now provides a rationale for treating this entity with a modified myeloma protocol based on anti-CD38 agents, cyclophosphamide, proteasome inhibitors, and dexamethasone [139]. If no monoclonal plasma cell population can be identified or the paraprotein is of the IgM subtype, the patient should be investigated for B-cell clones using computed tomography, PET scans, and flow cytometry of the peripheral blood. Accordingly, the therapy should be B-cell-directed using anti-CD20 agents, alkylating substances, and dexamethasone as single drugs or in combination. In view of the continued scarcity of systematic trials, the decision to treat an individual patient should be based on the severity of the disease, decline in kidney function, performance status, and patient preferences. Treatment response can be monitored by hematologic parameters since the hematologic response precedes and determines the renal response, as reported in AL amyloidosis and Ig-deposition disease [140,141].

### 7.4. Renal Amyloidosis as Cause of the Nephrotic Syndrome

AL amyloidosis is part of the MGRS spectrum and is a frequent cause of nephrotic syndrome. However, other rare forms of amyloidosis unrelated to monoclonal disorders may also affect the kidneys and cause nephrotic syndrome and chronic kidney disease. Amyloidoses constitute a heterogeneous group of diseases spanning the entire spectrum of clinical manifestations from local disease to widespread life-threatening systemic disease. Organ involvement varies from type to type, with cardiac involvement being the main life-limiting manifestation. Immunoglobulin-associated (AL)amyloidosis is by far the most frequent amyloidosis affecting the kidneys (>85% of all cases), followed by AA (7%), ALECT2 (2.7%), and fibrinogen A alpha chain (1.6%) amyloidosis. Other subtypes (ALys, ATTRv, AApoAI, AII, AIV, AGel, and ACys) each represented less than 1% of the sampled population [142,143]. Of the currently known 42 types of amyloidosis, 16 affect the kidneys (marked green in Table 14). Congo red staining of the renal biopsy samples identifies the typical birefringence of amyloid deposits and confirms the diagnosis of amyloidosis. However, subtyping depends on careful immunofluorescence studies and, in futile cases, mass spectrometry at international expert centers. With the exception of certain patients with cardiac ATTR amyloidosis, in whom a diagnosis can be established using bone scintigraphy, echocardiography, and serum studies to rule out monoclonal gammopathy, the diagnosis of amyloidosis requires biopstic confirmation.

Once the subtype of amyloidosis is established, further diagnostic measures may be required to identify the underlying disease. For instance, AA amyloidosis results from chronic inflammatory states such as chronic viral, bacterial, or helminthic infections but may also be related to autoimmune diseases such as rheumatoid arthritis or autoinflammatory syndromes such as familial Mediterranean fever. The underlying cause then informs therapy such as antimicrobial or antiviral therapy, immunosuppression, or anti-inflammatory drugs. In other hereditary forms, liver transplantation may remain the only curative option.

Recent scientific efforts foreshadow an intriguing expansion of the therapeutic arsenal for treating amyloidosis: inhibition of precursor production, prevention of plaque formation, and dissolution of amyloid plaques in tissues. Antisense oligonucleotides and siRNA techniques are being investigated to reduce the expression of defective precursor proteins and prevent aggregate formation to circumvent liver transplantation. Outside the renal context, these endeavors have already allowed the approval of inotersen, patisiran, and vutrisiran for the treatment of polyneuropathic forms of ATTR amyloidosis. Novel therapies have been designed to prevent amyloid plaque formation and misfolding (such as the tetramere-stabilizer tafamidis in ATTR amyloidosis). Amyloid plaque formation in Alzheimer’s has been successfully blocked by antibodies directed against protofibrils; some of these antibodies have already been approved by the FDA. Monoclonal antibodies that target amyloid plaques and induce a cellular inflammatory response are currently under clinical investigation for the treatment of AL-(birtamimab/NEOD001, anselamimab/CAEL-101) and ATTR amyloidosis (ALXN2220) with potential application in the renal context. The initial euphoria around this class of drugs was blunted by the results of the VITAL trial, demonstrating a lack of efficiency [144]. The AFFIRM-AL trial (NCT04973137) is underway to investigate if a subset of severely affected patients may benefit from the intervention. It is possible that the follow-up times were too short to detect meaningful differences in mildly or moderately affected individuals. With the primary outcome measure of time to all-cause mortality, the trial primarily addressed the efficacy of the novel agent in cardiac AL amyloidosis, which determines prognosis. Whether the results can be extrapolated to renal endpoints, particularly the reduction in proteinuria needs to be clarified in future trials and real-world experience. As indicated in Table 15, many trials involve an attempt to establish approved anti-myeloma agents in the treatment of systemic AL amyloidosis, while amyloidosis-specific drugs or protocols addressing AA- or other forms of amyloidosis (with the exception of ATTR) are exceedingly rare.

Interestingly, a trial (NCT06420167) of dapagliflozin aims to assess the effects of SGLT2 inhibitors on proteinuria in patients with AL amyloidosis. In light of recent successes in treating patients with severe autoimmune conditions with CAR-T-cells [145], initial attempts to treat refractory AL amyloidosis with the investigational CAR-T-cell preparation FKC288 are underway.

**Table 14:** Complete list of known subtypes of amyloidosis. The types with renal involvement are highlighted in green.

Name/Abbreviation	Etiology	Amyloidogenic Protein	Kidney Involvement
AL/AH	acquired	Immunoglobulin light or heavy chains	Yes
AA	acquired/hereditary	Serum amyloid A (SAA)	Yes
ATTRv	hereditary	Mutant transthyretin	Yes (PU in 1/3 of all patients, ESRD in 10%) [146]
ATTRwt	acquired	Wild-type transthyretin	Yes
ALect2	unknown	Leucocyte chemotactic factor 2 (LECT2)	Yes
AGel	hereditary	Gelsolin	Yes
AApoAI	hereditary	Apolipoprotein AI	Yes
AApoAII	hereditary	Apolipoprotein AII	Yes
AApoAIV	acquired/hereditary	Apolipoprotein AIV	Yes
AApoCII	hereditary	Apolipoprotein CII	Yes
AApoCIII	hereditary	Apolipoprotein CIII	Yes
AFib	hereditary	Fibrinogen A alpha chain	Yes
Aß2M	iatrogenic	Beta-2 microglobulin	Yes
ALys	hereditary	Lysozyme	Yes
ACal	malignant	Calcitonin	Yes
Aß2m	hereditary, iatrogenic	Beta-2-microglobulin	no
ASom	malignant	Somatostatin	no
AGluc	malignant	Glucagon	no
AGLP1	iatrogenic	Glucagon-like peptide analog	no
APTH	acquired, malignant	Parathyroid hormone	no
AIns	iatrogenic	Insulin	no
APro	acquired, malignant	Prolactin	no
AIAPP	acquired, malignant	Islet amyloid Amylin	no
AANP	acquired	Atrial natriuretic peptide	no
AKer	hereditary	Keratoepithelin	no
Abeta	acquired/hereditary	Amyloid precursor protein (APP)	no
APrP	acquired/hereditary	Prion protein (PRP)	no
ABri/ADan	hereditary	BRI gene product	no
ACys	hereditary	Cystatin C	no
ATMEM106B	acquired	Transmembrane 106B (TMEM106B)	no
ASPC	acquired	Lung surfactant protein	no
AIL1RAP	iatrogenic	Interleukin 1 receptor antagonist protein	no
LGMD D3	hereditary	Human heterogeneous ribonucleoprotein D-like (hnRNPDL)	no
ANO5	hereditary	Anoctamin5	no
DYSF	hereditary	Dysferlin	no
ALac	acquired	Lactoferrin	no
AOAAP	malignant	Odontogenic ameloblast-associated protein	no
ASem1	acquired	Semenogelin 1	no
AMed	acquired	Lactadherin	no
ACor	acquired	Corneodesmosin	no
AEnf	iatrogenic	Enfuvirtide	no
ACatK	malignant	Cathepsin K	no
AEFEMP1	acquired	Epithelial growth factor (EGF)-containing fibulin-like extracellular matrix protein 1 (EFEMP1)	no

**Table 15:** Selected trials as indexed at clinicaltrials.gov (July 2024) for AL and AA amyloidosis. ATTR amyloidosis trials have been omitted.

Clinicaltrials.gov Identifier	Entity	Drug
NCT06397001	AA	nL-SAA1-01 (antisense oligonucleotide)
NCT05199337	AL	ZN-d5
NCT05145816	AL	Belantamab Mafodotin
NCT02312206	AL	Birtamimab
NCT04973137	AL	Birtamimab
NCT06342466	AL	Pomalidomid
NCT05451771	AL	Venetoclax
NCT03236792	AL	Ixazomib
NCT05898646	AL	Daratumumab maintenance
NCT01789242	AL	Carfilzomib
NCT04298372	AL	Lenalidomid
NCT05066607	AL	Isatuximab
NCT03618537	AL	Ixazomib maintenance
NCT06420167	AL	Dapagliflozin
NCT05978661	AL	FKC288 (CAR-T-cells)
NCT06158854	AL	ABBV-383 (BCMA-directed)
NCT06292780	AL	Linvoseltamab (BCMA-bispecific)
NCT05839626	AL	SAR445514 (NKp46/CD16-based BCMA-targeted NK cell engager)
NCT05652335	AL	JNJ-79635322, a tri-specific antibody (BCMA-GPRC5D-CD3)

## 8. Diabetic Kidney Disease

Diabetic kidney disease (DKD) is the leading cause of CKD and ESKD worldwide and affects about 30% of patients with type 1 diabetes (TDM1) and up to 40% of patients with type 2 diabetes (TDM2) [147]. Estimating the prevalence of DKD is difficult as the diagnosis is usually based on renal dysfunction in association with pre-existing diabetes, but most of the time without histological confirmation [147,148]. Non-renal diagnoses as the cause of kidney disease in patients with diabetes are not rare and often overseen [149], and the real prevalence of DKD might even be overestimated. Hyperglycemia, impaired insulin receptor signaling, advanced glycation end-product toxicity, and glomerular inflammation can directly affect podocyte function, but hemodynamic factors leading to single-nephron glomerular hyperfiltration also contribute to the pathophysiology of diabetic nephropathy. The phenotypes of DKD are heterogeneous, with presentation patterns reaching from non-proteinuric DKD to diabetic kidney disease with heavy proteinuria and nephrotic syndrome. Due to the large number of diabetic patients, DKD is the most frequent cause of nephrotic syndrome. Onset of disease is usually gradual and primary glomerular disease is more likely in a setting of acute onset. 

In recent years, various potential urinary markers for DKD have been identified [150]. Proteomics markers such as urinary CKD273 [151], a proteome-based classifier consisting of 273 peptides, are promising candidates for diagnostic and prognostic purposes in DKD, even at the very early stages that cannot be diagnosed with traditional markers such as albuminuria and serum creatinine or eGFR decline [152]. Therapeutic advances improved individual renal and cardiovascular prognosis, mainly through the evolution of SGLT2 Inhibitors in addition to RAAS inhibition [87,153,154]. Further therapeutic developments included the introduction of nsMRAs (Finerenone) with evidence for renal protection [155,156,157] as well as new data for renal benefits with GLP1-R agonists [158]. The main therapeutic goal is not only to inhibit renal progression but also cardiovascular protection. In contrast to all previous glucose-lowering therapies, the new substances mentioned above are effective in addressing this purpose by significantly improving cardio-renal-metabolic prognosis.

## 9. Fibrillary Glomerulonephritis

Fibrillary glomerulonephritis (FGN) is a rare glomerular disease defined by glomerular deposition of Congo red-negative, randomly oriented straight fibrils that lack a hollow center and, in most cases, stain with antisera to immunoglobulins by immunofluorescence [159]. It should be distinguished from the rare immunotactoid glomerulopathy, which is characterized by the deposition of larger and stacked microtubules composed of mostly monoclonal proteins [160]. FGN is found in less than 1% of native kidney biopsies, and patients are usually between 45 and 65 years old. FGN has long been classified as idiopathic, but there is growing evidence of an association with autoimmune diseases, cancer, and hepatitis [161,162,163]. DnaJ homolog subfamily B member 9 (DNAJB9) has recently been identified as a novel tissue biomarker of FGN [164,165,166]. DNAJB9 belongs to a family of proteins that functions as “co-chaperones” to heat-shock protein 70 (hsp-70). It is expressed in all healthy tissues, is localized to endoplasmic reticulum (ER), and is upregulated by ER stress, nitric oxide, and other inflammatory mediators. DNAJB9 immunohistochemistry has a 98% sensitivity and >99% specificity for FGN [164] and can be considered as a highly specific diagnostic tool in the still challenging diagnosis of FGN. It provides the diagnosis also in areas without the availability of electron microscopy and discriminates FGN from other rare glomerular diseases with similar patterns and in cases with concurrent other glomerular diseases [164]. DNAAJB9 staining has become the gold standard in the diagnosis of FGN. The pathophysiology of the accumulation of DNAJB9 is still unclear. There is no evidence of mutations in the DNAJB9 gene or structural DNAJB9 alterations in FGN cases [165] and no glomerular transcriptional upregulation of DNAJB9 [167]. The source of the DNAJB9 overproduction is therefore unknown. There is a 4-fold higher abundance of serum DNAJB9 in FGN patients when compared to controls [168]. Serum DNAJB9 levels accurately predicted FGN with moderate sensitivity (67%), high specificity (98%), and a positive and negative predictive value of 89% and 95%, respectively [168]. The glomerular deposits of immunoglobulins and fibrils of DNAJB9 may cause indirect podocyte injury and may eventually cause nephrotic syndrome. Absolute serum DNAJB9 concentration differences among groups with FGN and other renal diseases are modest and have considerable overlap. Identification of elevated serum concentrations of DNAJB9 in patients with FGN has the potential to enhance the non-invasive diagnosis of FGN, but currently, a renal biopsy is still required to diagnose FGN. 

There is a paucity of evidence-based therapeutic recommendations. Most patients with preserved kidney function and non-nephrotic proteinuria are treated with RAAS inhibition and maybe SGLT2 inhibitors. Immunosuppressive therapy could be considered in patients with high proteinuria or even nephrotic syndrome, but data from case series are not very encouraging [161,169]. It is recommended to control and treat possible underlying diseases. A pilot study with rituximab in FGN should promising results [170].

## 10. Drug-Induced Nephrotic Syndrome

The most common manifestation of drug-induced nephrotoxicity is acute kidney injury, mainly as a result of acute interstitial nephritis or acute tubular necrosis. But medication can affect every part of the nephron, including glomerular damage [171]. Certain drugs are associated with new onset or worsening of proteinuria and even nephrotic syndrome by induction of glomerular disease. The histologic patterns usually include minimal change disease, membranous nephropathy, focal segmental glomerulosclerosis, vasculitis, drug-induced lupus, and thrombotic microangiopathy.

Drugs associated with proteinuria and nephrotic syndrome are listed in Table 16.

## 11. Conclusions

There has been an evolution in our knowledge of primary glomerular diseases and nephrotic syndromes. Histological patterns are not sufficient for diagnosis and do not guide treatment decisions but lead to a pathway of further targeted investigations. Advances in our understanding of the pathogenesis also guide new proposed classifications of glomerular diseases [172]. New biomarkers led to easier and more accurate diagnoses and more targeted therapeutic decisions (e.g., PLA2-R-ab and anti-nephrin-ab). More precise phenotyping of the diseases and the use of modern nephrogenetic possibilities not only help to improve treatment but also may be guidance to avoid unnecessary exposure to potentially toxic immunosuppression. The treatment landscape is becoming wider, and there is a pipeline of promising new therapeutic agents with more sophisticated approaches.

## Figures and Tables

**Table 1:** Known MN target antigens (adapted from [15]).

Target Antigen	Podocyte Expressed	TM vs. Secreted	Clinical/Disease Association	Distinctive Histopathologic Features	Incidence
PLA2R	Yes	TM	None	Global, granular, subepithelial deposits; IgG4 predominant	55%
THSD7A	Yes	TM	Malignancy	Similar to PLA2R	2%
NELL1	No	secreted	Malignancy, Drugs, AID	IgG1 predominant, deposits may be a segmental or incomplete-loop pattern	10%
SEMA3B	Yes	secreted	Pediatric	IgG1 predominant, may have additional mesangial deposits; TBM deposits may be present	2%
PCDH7	No	TM	Older	C3 absent or weak	2%
HTRA1	Yes	secreted	None	IgG4 predominant, similar to PLA2R	<1%
NTNG1	Yes	secreted	None	IgG4 predominant, similar to PLA2R	<1%
EXT1/EXT2	No	TM	AID, Lupus	IgG1 predominant, IgA, IgM often present, mesangial deposits, may coexist with class III/IV lupus	7%
NCAM1	No	TM	Lupus	Similar to EXT1/EXT2	2%
TGFBR3	Yes	TM	Lupus	Similar to EXT1/EXT2	
CNTN1	No	secreted	CIDP	IgG4 predominant	1%
FAT1	Yes	TM	HSCT	TBM deposits can be present	1%
NDNF	Yes	secreted	Syphilis	Lumpy deposits, superficial hump-like by EM, IgG1	1%
PCSK6	No	secreted	NSAID	IgG1 and 4	2%

CIDP, chronic inflammatory demyelinating polyradiculoneuropathy; CNTN1, contactin 1; EM, electron microscopy; EXT, exostosin; FAT1, protocadherin FAT1; HSCT, hematopoietic stem cell transplant; HTRA1, serine protease HTRA1; MN, membranous nephropathy; NCAM1, neural cell-adhesion molecule 1; NDNF, neuron-derived neurotrophic factor; NELL1, neural epidermal growth factor-like protein 1; NSAID, nonsteroidal anti-inflammatory drug; NTNG, netrin G1; PCDH7, protocadherin 7; PCSK6, proprotein convertase subtilisin/kexin type 6; PLA2R, M-type phospholipase A2 receptor; SEMA3B, semaphorin 3B; TBM, tubular basement membrane; TGFBR3, transforming growth factor beta receptor 3; THSD7A, thrombospondin type-I domain-containing 7A; TM, transmembrane; AID, autoimmune disease.

**Table 2:** Risk stratification of MN by clinical criteria for assessing the risk of progressive loss of kidney function (adapted from [23]).

Low Risk	Moderate Risk	High Risk	Very High Risk
eGFR60 mL/min/1.73 m^2^ Proteinuria <3.5 g/d Serum albumin >3 g/dL Or eGFR 60 mL/min/1.73 m^2^ and 50% reduction in proteinuria in <6 months under therapy with RAASi	eGFR > 60mL/min/1.73 m^2^ Proteinuria > 3.5 g/d and no decline > 50% after 6 month of RAASi And Not fullfilling high-risk criteria	eGFR < 60mL/min/1.73 m^2^ and/or proteinuria > 8 g/d for >6 month Or eGFR > 60 mL/min/1.73 m^2^, Proteinuria > 3.5g/d and no decline > 50% after 6 months of RAASi and at least one of the following: -serum albumin < 2.5g/dL -anti-PLAR2ab > 50 RU/mL -urinary a1-microglobulin > 40 µg/min -urinary IgG > 1 µg/min -urinary b2 microglobulin > 250 mg/d -selectivity index > 0.2	Life-threatening nephrotic syndrome or Rapidly deteriorating kidney function

**Table 3:** Landmark trials in primary membranous nephropathy.

Study	N	Intervention	Results
GEMRITUX 2017 [25]	77	Two doses of 375 mg/m^2^ RTX vs. SOC	RTX + SOC: 65% at 17 months (CR 19%) SOC: 34% at 17 months (CR 3%)
MENTOR 2019 [26]	130	1000 mg RTX d1 + d14, repeated at 6 months if partial response vs. CyA	RTX: 62% at 18 months (CR 28%) CyA 33% (CR 2%)
RI-CYCLO 2021 [27]	74	1000 mg RTX d1 + d14 vs. 6 m cyclic regimen GC alternated with CYP every other month	RTX: 66% at 18 months (CR 31%) CYP-GC: 79% at 18 months (CR 21%)
STARMEN 2021 [28]	86	6 m cyclic regimen with GC alternated with CYP every other month vs. Tac 0.05 mg/kg/d first 6 months, followed by RTX 1 g	CYP-GC: 84% at 18 months (CR 44%) Tac-RTX: 53% at 18 months (CR 16%)

SOC: standard of care. CYP: cyclophosphamide. GC: glucocorticoids. CR: complete remission. CyA: cyclosporine A, Tac: tacrolimus. RTX: rituximab, m: month, d: day.

**Table 4:** Histological finding on the first biopsy in patients with lupus nephritis, adapted from [33].

Histological Finding on 1st Biopsy	
Class I	0–6%
Class II	1–20%
Class III	10–25%
Class IV	35–60%
Class V	5–30%
Class VI	<5%

**Table 5:** Causes of secondary IgAN [76].

Group	Disease
Gastrointestinal and liver diseases	Inflammatory bowel disease, celiac disease, cirrhosis
Infection	HBV, HCV, HIV, tuberculosis, leprosy
Autoimmune diseases	Ankylosing spondylitis, rheumatoid arthritis, Sjögren syndrome
Malignancy	Lung cancer, renal cell carcinoma, non-Hodgkin and Hodgkin lymphoma, IgA myeloma
Respiratory tract	Sarcoidosis, bronchioloitis obliterans, pulmonary hemosiderosis, cystic fibrosis, pulmonary fibrosis
Skin	Dermatitis herpetiformis, psoriasis

**Table 6:** Supportive Care in IgAN.

Supportive Care in IgAN
Blood pressure control
Reduction in proteinuria with RAAS-inhibitors and SGLT2i
Treatment of dyslipidemia
Lifestyle modification (dietary sodium restriction, smoking cessation, weight control, exercise)

**Table 7:** Selected trials of investigational drugs in IgAN as indexed at clinicaltrials.gov.

Investigational Agent	Mechanism of Action	Clinicaltrials.gov Identifier
Complement inhibitors		
Narsoplimab	MASP-2 inhibition (lectin pathway)	NCT02682407, NCT03608033
Iptacopan	Complement factor B inhibitor (alternative pathway)	NCT03373461, NCT04578834
Vemircopan	Complement factor D inhibitor (alternative pathway)	NCT05097989
Cemdisiran	Complement factor 5 inhibitor (common pathway)	NCT03841448
Ravulizumab	Complement factor 5 inhibitor (common pathway)	NCT06291376, NCT04564339
Pegcetacoplan	Complement factor 3 inhibitor (common pathway)	NCT03453619
Avacopan	C5aR1/inhibition of anaphylatoxin (common pathway)	NCT02384317
BAFF/APRIL inhibitors		
Sibeprenlimab	Antibody targeting APRIL	NCT05248646
Zigakibart	Antibody targeting APRIL	NCT05852938
Atacicept	Neutralizes activity of APRIL and BAFF	NCT04716231
Telitacicept	Neutralizes activity of APRIL and BAFF	Coming
Povetacicept	Neutralizes activity of APRIL and BAFF	NCT05732402
Endothelin-1 antagonists		
Atrasentan	Antagonist of endothelin A receptor	NCT05834738
Sparsentan	Inhibition of angiotensin II and endothelin A receptors	NCT05003986 (children)

**Table 8:** Causes for secondary I-MPGN.

Disease Group	Disease
infectious diseases	hepatitis C (with or without cryoglobulinemia)
	hepatitis B
	HIV, EBV
	bacterial endocarditis
	visceral abscess
	ventriculoatrial shunt infection
	protozoa infection
	mycoplasm infection
	tuberculosis
	brucellosis
	malaria
	schistosomiasis
	echinococcosis
autoimmune/rheumatologic disorder	systemic lupus erythematosus
	cryoglobulinemia
	sjögren’s syndrome
	rheumatoid arthritis
	mixed connective tissue disease
monoclonal gammopathies and neoplasia	monoclonal gammopathy of unknown significance
	chronic lymphocytic leukemia
	lymphoma
	leukemia
	multiple myeloma
	Waldenstrom macroglobulinemia
	carcinoma
	malignant melanoma
other	alpha 1 antitrypsin deficiency
	sickle cell disease
	thrombotic microangiopathy
	transplant glomerulopathy

**Table 9:** Diagnostic workup in C-MPGN at initial evaluation.

Complement Analysis	CH50
	APH50
	C3
	C4
	C3d
	sC5b-9
	factor H
	factor I
	factor B
autoantibodies	C3NeF
	anti-factor B
	anti-C3 convertase
	anti-factor H
genetic analysis	factor H
	factor I
	CFHR1-5
	MCP/CD46
	C3
exclusion of differential diagnosis	ANA
	ANCA
	anti-GBM-antibodies
	HIV serology
	HBV serology
	HCV serology
	serum and urine protein electrophoresis

**Table 10:** Causes of secondary MCD [121].

Group	Disease
Drugs	NSAID, salazopyrin, D-penicillamine, mercury exposure, gold, tiopronin, lithium, tyrosine-kinase inhibitors
Malignancies	Hodgkin lymphoma, non-Hodgkin lymphoma, leukemia, multiple myeloma
Infections	Syphilis, tuberculosis, mycoplasma, ehrlichiosis, hepatitis C, echinococcus, borreliosis
Allergy	Fungi, pollen, dust, bee stings, cat fur, food allergens
Autoimmune disorders	systemic lupus erythematosus, type 1 diabetes mellitus, myasthenia gravis, autoimmune pancreatitis, celiac disease

**Table 11:** Causes of secondary FSGS (adapted from [24]).

Secondary to Alterations of Glomerular Epithelial Cells	
Viral infections	HIV, CMV, Parvovirus B12, EBV, HCV, Hemophagocytic syndrome, SARS-CoV-2
Drug-induced	Direct-acting antiviral therapy, mTOR inhibitors, Calcineurin inhibitors, anthracyclines, heroin, lithium, interferon, anabolic steroids, NSAIDs
Secondary to adaptive changes with glomerular hypertension	
Reduced nephron number	Reflux nephropathy, renal dysplasia, oligomeganephronia, sickle cell disease, age-related FSGS
Normal nephron number	Obesity-related glomerulopathy, primary glomerular diseases, systemic conditions (e.g., diabetic nephropathy, hypertensive nephrosclerosis)

**Table 12:** Definition of remission, relapse, resistance, and dependence for MCD and FSGS. PCR: protein-creatinine ratio. (Adapted from [24]).

Complete remission	Reduction in proteinuria to ˂0.3 g/d or PCR < 300 mg/g, stable serum creatinine and serum albumin > 3.5 g/dL
Partial remission	Reduction in proteinuria to 0.3g–3.5/d or PCR < 300–3500 mg/g and a decrease > 50% from baseline
Relapse	Proteinuria > 3.5g/d or PCR > 3500 mg/g after complete remission has been achieved or an increase in proteinuria > 50% in patients who had undergone partial remission
Steroid-resistant MCD/FSGS	Persistence of proteinuria > 3.5 g/d or PCR > 3500 mg/g with <50% reduction from baseline despite prednisone 1 mg/kg/d for >16 weeks
Steroid-dependent MCD/FSGS	Relapse occurring during or within 2 weeks of completing glucocorticoid therapy
Frequently relapsing MCD	Two or more relapses per 6 months

**Table 13:** Renal manifestations of monoclonal gammopathies may produce heavy proteinuria and nephrotic syndrome.

Manifestation	Albuminuria/Nephrotic Syndrome	Ig-Subtype Association
AL/AH/AHL amyloidosis	+++/> 60%	lambda >> kappa
Monoclonal immunoglobulin deposition disease MIDD (LCDD, LHCDD, HCDD)	++/~ 20%	kappa >> lambda
Proliferative glomerulonephritis with monoclonal immunoglobuline deposits (PGNMID)	+++/~ 50%	2/3: no detectable circulating monoclonal protein. 1/3: detectable, often IgG3kappa
Type 1 Cryoglobulinemic glomerulonephritis	++/~ 35–40%	renal: IgG >> IgM articular: IgG3
Light-chain proximal tubulopathy	+(+)/rare	kappa >> lambda
C3 glomerulopathy with monoclonal gammopathy	++/~ 40%	
Thrombotic microangiopathy with monoclonal gammopathy	+(+)/~40%	
Monoclonal immunotactoid glomerulonephritis	+++/~70%	lambda >> kappa
Light chain crystalline podocytopathy	++/~ 30%	kappa >> lambda

+ corresponds to low grade proteinuria, ++ corresponds to medium range proteinuria (>100 mg/dL), and +++ to large nephrotic proteinuria (> 300 mg/dL).

**Table 16:** Drugs associated with proteinuria and nephrotic syndrome.

Drugs	Associated Disease
IFN-*α* and-*β*	MCD, FSGS, MN
IFN-*γ*	FSGS
bisphosphonates (e.g., pamidronate, zoledronate)	MCD, FSGS
anabolic steroids	FSGS
lithium	MCD, FSGS, MN
NSAIDs	MCD, MN
cyclooxygenase-2 inhibitors	MCD, MN
mTOR inhibitors (e.g., sirolomis, everolimus)	FSGS
antibiotics (e.g., Ampicillin, rifampicin, cefixime)	MCD
tamoxifen	MCD
penacillamine	MCD, MN
bucillamine	MCD, MN
etanercept	MCD
gold	MCD, MN
methimazole	MCD
enalapril	MCD
mercury	MCD
heroin	FSGS
fluconazole	MN
probenecid	MN
captopril	MN
mercury	MN
clarithromycin	MN
VEGF pathway inhibitors (e.g., bevacizumab, Sunitinib/Sorafenib, Aflibercept, Ramucirumab)	MCD, FSGS, MN
Src family kinase inhibitors (e.g., dasatinib)	MCD, FSGS, endotheliosis
EGFR pathway inhibitors (e.g., erlotinib, gefitinib, cetuximab, panitumumab	MCD, MN

NSAIDs: non-steroidal anti-inflammatory drugs, COX: cyclooxygenase, mTOR mammalian: target of rapamycin, MCD: Minimal change disease, FSGS: focal segmental glomerulosclerosis, MN: membranous glomerulonephritis.
